# The Carbon Footprint of Valencia Port: A Case Study of the Port Authority of Valencia (Spain)

**DOI:** 10.3390/ijerph17218157

**Published:** 2020-11-04

**Authors:** Víctor Cloquell Ballester, Vanesa G. Lo-Iacono-Ferreira, Miguel Ángel Artacho-Ramírez, Salvador F. Capuz-Rizo

**Affiliations:** 1 Department of Engineering Projects, Valencia Campus, Universitat Politècnica de València, Camino de Vera, s/n, E-46022 Valencia, Spain; vacloque@dpi.upv.es (V.C.B.); miarra@dpi.upv.es (M.Á.A.-R.); scapuz@dpi.upv.es (S.F.C.-R.); 2 Department of Engineering Projects, Alcoy Campus, Universitat Politècnica de València, Plaza Ferrándiz y Carbonell, s/n, E-03690 Alcoy, Spain

**Keywords:** GHG, emissions, maritime transport, energy consumption, environmental performance

## Abstract

Maritime transport is responsible for 13% of the Greenhouse Gases (GHG) emissions of the transport sector. Port authorities, terminals, shipping companies, and other stakeholders have joined efforts to improve this sector’s environmental performance. In Spain, the Ministry for Ecological Transition and Demographic Challenge has developed a methodology to assess the carbon footprint. This methodology has been adapted to ports and applied to processes under the Port Authority of Valencia’s umbrella achieving scopes 1, 2, and 3. The results highlight that ship traffic, within the port, of containers and cruises (categorized in scope 3) had a major impact on the carbon footprint. Buildings lighting managed by the terminals has a significant effect on scope 2. Diesel consumption shares with gasoline consumption the primary representation in scope 1. The carbon footprint between 2008 and 2016 was maintained, although traffic in the port increased by 24% during this period. The results show a decrease of 17% when emissions are compared using the base year’s emissions factors to avoid external factors. Future projects that include self-consumption or renewable energy policies seem to be the next step in a port that shows good results but still has room for improvement in activities of scope 3.

## 1. Introduction

The Valencia Port, with over 5000 twenty-foot equivalent units (TEUs) a year, is the 6th largest port of container traffic in Europe and the largest in the Mediterranean Sea [1,2]. Five thousand ships, including container ships, cruise, and ferries, operate each year in this Spanish port. The port authority’s commitment led to the implementation of an environmental management system verified in EMAS and ISO 14,001 in 2008 and constant evolution since then [3,4].

The carbon footprint is a popular indicator applied for processes, products and organizations, maritime activities, and ports [5,6,7,8]. The Valencia Port applied it to emissions resulting from cruise and sport boats and big 400 m long container ships that can take almost 15,000 TEU [9].

There are many standard methodologies to assess the carbon footprint. The most widely known are ISO 14067:2018 [10] and PAS 2050 [11], both based on a life cycle thinking that considers different levels of detail or scopes.

The transport sector is responsible for 29% of all energy consumption globally and, therefore, for a similar amount of GHG emissions [12,13]. Maritime transport is responsible for 13% of the total GHG emissions [14]. Maritime transport also emits other harmful gases such as SOx and NOx [15,16,17,18]. However, there are significant benefits when choosing maritime transport over different types of transport. When maritime transport is not possible, rail transport might be a more sustainable choice. For example, the transport of goods by road presents a higher number of accidents, generates traffic congestion, and is responsible for more than 60% of the GHG emissions [19,20].

Ports are essential infrastructures for economic growth. Providing accurate information also helps involve stakeholders in the projects developed to improve ports’ environmental performance [21,22]. Port terminals have made a significant effort to assess their carbon footprint [23,24,25,26,27,28]. Although the methodologies applied to vary significantly and, often, only the direct emissions are considered [29]. There are no studies that address all three scopes under a structured methodology.

The port of Valencia, managed by its Port Authority, has made a significant effort in the past years, developing projects and initiatives seeking to improve their environmental performance. This research assesses the carbon footprint of one of the main ports in Europe, Valencia port, applying a standardized methodology and considering direct and indirect emissions (scopes 1, 2, and 3). This organization is the first of its kind that assesses and certifies according to ISO 14064:2018 its carbon footprint, including scope 3.

This study aims to present and analyze the carbon footprint assessment carried by the Authority Port of Valencia. This paper is structured in several sections. Section 2 describes the methodology and data characteristics. Results are presented in Section 3, including a parallel assessment for comparative porpoises. Section 3 also shows the discussion of results, and Section 4, the conclusions of the study.

## 2. Methodology and Data

Valencia’s port authority (Spain) ’s carbon footprint was calculated under the Life Cycle Assessment (LCA) method by setting the system boundaries, defining the greenhouse gases, establishing the calculation formula, and interpreting the results obtained. The Guide for the Calculation and Management of the Carbon Footprint in Port Facilities by Levels was applied [30]. This guide follows the International Panel on Climate Change (IPCC) guidance.

### 2.1. Functional Unit, System Boundaries, Scope Definition, and Greenhouse Gases

**Figure 1:**
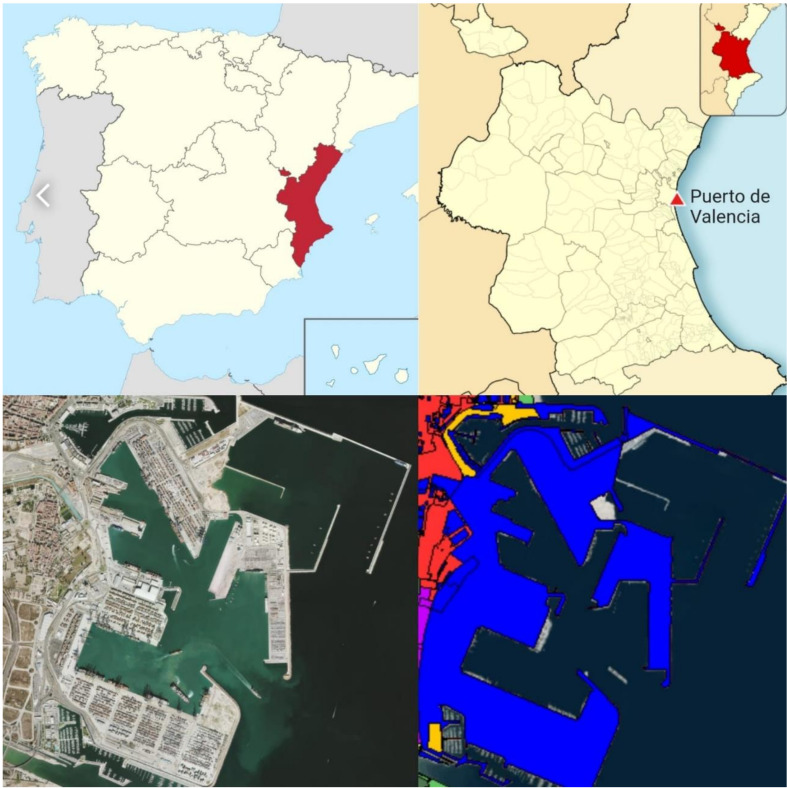
Port of Valencia location. Up left: Valencia region. Upright: Port location. Down left: Satellite image of the port. Downright, in blue, system under study. Source: [31].

The system under study was Valencia’s port authority, located in Valencia’s port (Spain; Figure 1). The management of the port uses tons of cargo managed as the base unit when assessing its activity. Therefore, and following the guide’s criteria [30], the cargo, measured in tons (t), was chosen as the study’s functional unit.

**Figure 2:**
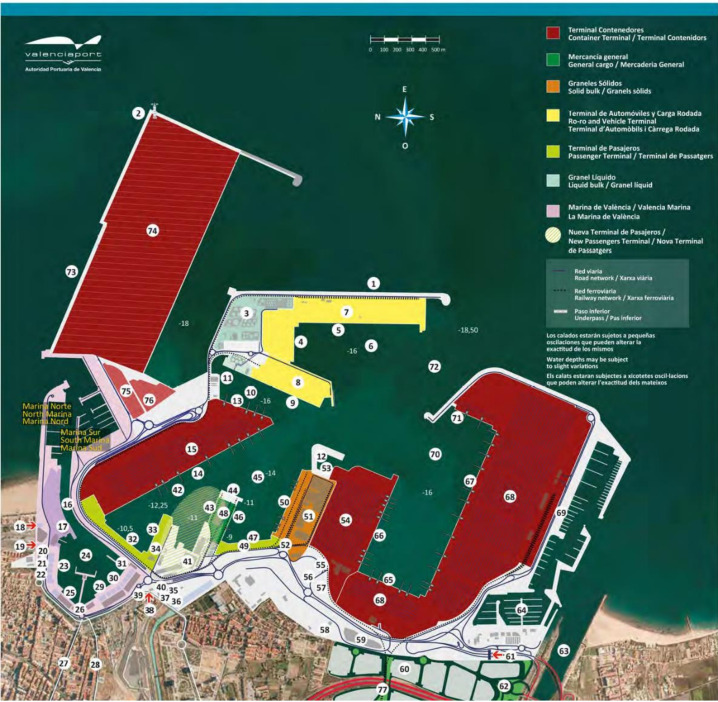
Port of Valencia. Source: [1]. Numerical legends: 1—East Breakwater, 2—Lighthouse, 3—Chemical and Oil Terminal, 4—Transversal East, 5—East Breakwater Quay, 6—East Dock, 7—Ro-ro and Vehicle Terminal 1, 8—Ro-ro and Vehicle Terminal 2, 9—North Quay (Xitá), 10—Xitá Dock, 11—Scrapyard Quay, 12—Port services (pilots, tug boats and mooring), 13—Llavera Quay, 14—Levante Quay, 15—Container Terminal 3, 16—Moveable bridge, 17—Veles e Vents building, 18—Access to Juan Carlos I Royal Marina, 19—Customs gate, 20—Customs Administration, 21—Foreign Health Department, 22—Valencia 2007 Consortium, 23—Customs Quay, 24—Inner Dock, 25—Grao Quay, 26—Clocktower building, 27—Avda. del Puerto, 28-Avda. Baleares, 29—Former Terminal Quay, 30—Nazaret Quay, 31—Fish Market, 32—Transversal Quay, 33—Poniente Quay, 34—Ferry Terminal/Passenger and Cruise Terminal, 35—Port Police, 36—Valencia port Foundation, 37—Port Authority of Valencia, 38—Nazaret gate, 39—Naval Command, 40—Plant Health Service, 41—Foreign Trade Inspection Centre, 42—Levante Dock, 43—North Turia Jetty Quay, 44—End Turia Jetty, 45—Turia Dock, 46—South Turia Jetty Quay, 47—Turia Quay, 48—General and bulk cargo, 49—Passenger Terminal, 50—South Quay, 51—Solid Bulk Terminal, 52—Spanish Customs Control Authority, 53—Technical and Nautical services Dock, 54—Container Terminal 2 (MSC), 55-PIF, 56—Harbourmaster’s Office, 57-Cold sage warehouses, 58—CPE Valencia, 59—Logistics warehouse, 60-Logistics Activities Area (ZAL), 61—South Access, 62—ZAL Access, 63—New Turia riverbed, 64—Royal Valencia Yacht Club, 65—Costa Quay, 66—Transversal Costa Quay, 67—Príncpie Felipe Quay, 68—Public Container Terminal 1, 69—Marine Civil Guard Building, 70—South Dock, 71—East Wuay, 72—Entrance channel, 73—North Extension Breakwater, 74—New Container Terminal, 75—Container depot 1, 76—Container depot 2. 77—Connection to national rail network.

The system boundaries were set to the area of influence of the port authority; this includes the offices of the port authority, stockyard machinery and activities, docking line machinery and activities, and ship traffic inside the port area (Figure 2).

Categories are applied to this study to assess better and analyze the results. The following three scopes were being considered:

- Scope 1 is defined by the direct emissions in the infrastructures, machinery, and vehicles of the port authority. Scope 1 includes all the processes under the direct control of the port authority that are not related to electricity consumption (scope 2). The emissions of these processes are direct emissions caused by the consumption of different fuels. For this reason, the categories in scope 1 are defined by the type of fuel.
- Scope 2 reflects the emissions associated with the electricity consumption of the infrastructures of the port authority. The port authority consumes electricity in two main areas under their own management: buildings and stockyard; although, there are some other small consumptions not included in both these areas. For this reason, scope 2 is organized into 3 categories: buildings, stockyard, and others. The category for building is divided into two subcategories: lighting and air conditioning.
- Scope 3 gathers the emissions of machinery, vehicles, and ships, including electricity consumption of third parties that operate within the area of influence of the port authority.

The categories in this scope are organized considering the management system of the port authority. This includes direct fuel consumption (diesel and gas), transport (ship traffic and land transport), and dealership electricity. Subcategories have been defined for a better description and analysis.

The categories defined that are included in the scope are described in Table 1.

The greenhouse gases considered were carbon dioxide (CO_2_), methane (CH_4_), and nitrous oxide (N_2_O) produced during the electricity generation and associated with fuel consumption (gas, gasoline, and diesel).

### 2.2. Carbon Footprint Assessment Method

The methodology defined by the Spanish Ministry for Ecological Transition and Demographic Challenge was followed [32], and scope 3 was introduced, as the original methodology does not include it. The port authority’s carbon footprint refers to the total greenhouse gas emissions from all the categories included in the previously defined scope. The formula applied for the calculation is as follows:

(1) $$CF=\overset{n}{\underset{i=1}{\sum }}GHGE_{i}=\overset{n}{\underset{i}{\sum }}{\left(E_{CO_{2}}+E_{CH_{4}}\cdot GWP_{CH_{4}}+E_{N_{2}O}\cdot GWP_{N_{2}O}\right)}_{i}$$

where *CF* stands for the carbon footprint, *GHGE* is greenhouse gas emissions, *E* represents emissions, *GWP* is the global warming potential of the gas identify as the subindex, and *i* identifies each category or subcategory, if any. Emissions for each greenhouse gas is assessed as follows:

(2) $$E_{g}=C_{i}\cdot EF_{i,g}$$

where *g* stands for each gas (*CO*_2_, *CH*_4_, and *N*_2_O), *C* stands for the consumption associated with each category, and *EF* represents the gas emission factor for the *i* category. Emission factors were obtained from the Spanish Ministry for Ecological Transition and Demographic Challenge [32,33]. Emissions factors for each year assessed and its source are described in Appendix A.

### 2.3. Data

The data used in this study for scope 1 and 2 were obtained from either the accounting system (invoices) or meters (fuel dispensers). Estimations were made for scope 3 based on the port’s activity registers and machinery and ships’ technical information.

Although the port’s environmental management system was implemented in 2008, it was improved in 2012, allowing annual data for energy consumption. The assessment was carried for 2008, 2010, 2012, 2013, 2014, 2015, and 2016.

## 3. Results and Discussion

**Figure 3:**
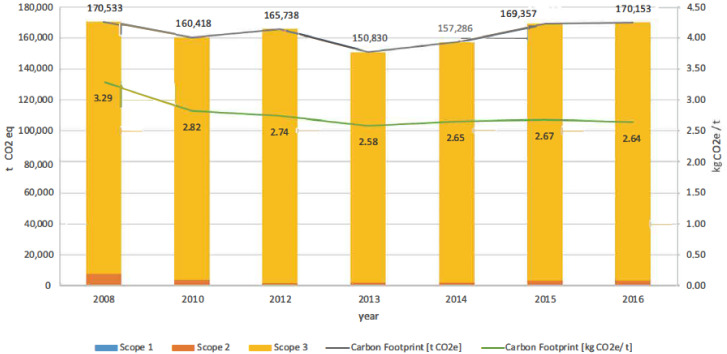
The carbon footprint of the port authority of Valencia (Spain) by year and category.

This section shows the results obtained for assessing the carbon footprint of the port authority of Valencia in the years 2008, 2010, 2012, 2013, 2014, 2015, and 2016 by scope (Figure 3). The green line (secondary axe) shows the evolution of the carbon footprint in kg CO_2_ eq by ton managed in the port. This value includes bulk cargo (liquid and solid), containerized and non-containerized goods, and fish catches and supplies.

The scope that improved the most was scope 2, reducing its carbon footprint by 57.78% between 2008 and 2016. Scope 1 had a significant improvement of 28.98%; however, the carbon footprint of scope 3 increased by 2.49%.

It should be highlighted that scope 3 was, by far, the scope with the highest representation in the overall results, with 97% on average during the period under analysis. Total emissions (grey line) only decreased by 0.22% between 2008 and 2016. However, it should be considered that the activity of the port increased by 24.33%, moving more than 64 million tons in 2016. As a result of several projects implemented to improve the process’s energy efficiency within the port [34], cargo’s carbon footprint decreased by 19.75%.

**Figure 4:**
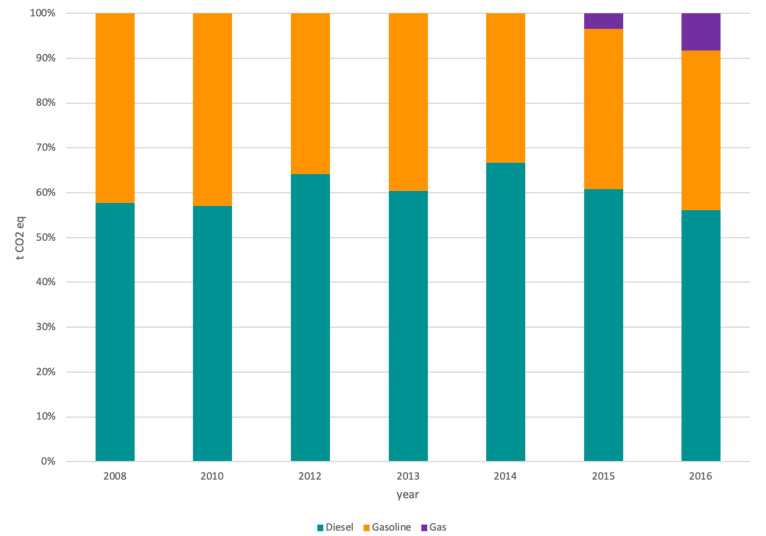
Categories breakdown for scope 1 by year.

**Figure 5:**
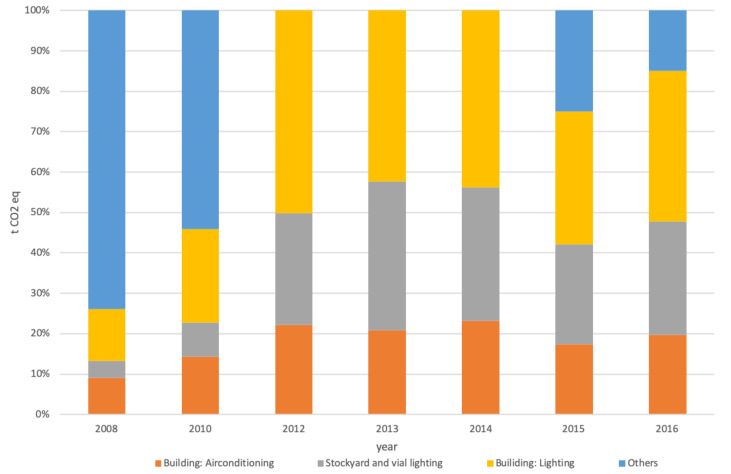
Categories breakdown for scope 2 by year.

**Figure 6:**
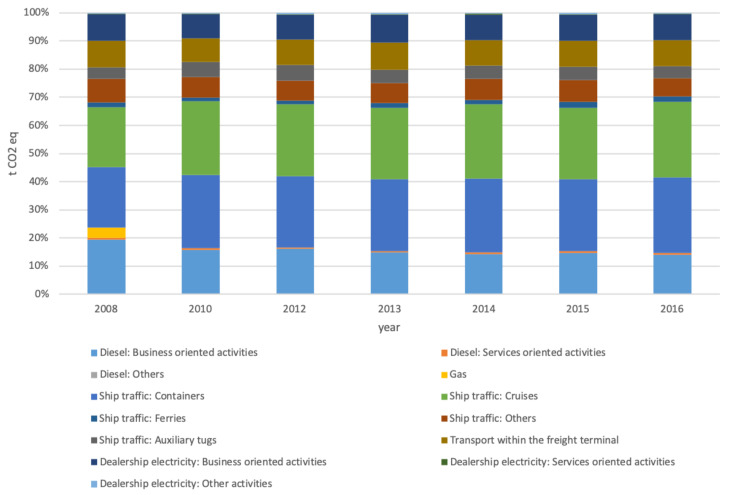
Categories breakdown for scope 3 by year.

Figure 4, Figure 5 and Figure 6 allow a more in-depth analysis by breaking down categories and subcategories of each scope.

The carbon footprint related to diesel consumption was the most significant, although gasoline can represent 40% of the total. Gas was introduced as a fuel for activities related to scope 1 in the last two years analyzed; this is why it did not appear in previous years. However, the impact was less than 10%.

Scope 2 results offered more variation than scope 1. It can be seen that the “others” category, including the consumption of electricity by industrial machinery, had a significant representation in the first two years of analysis and appeared again in the last two. This subcategory includes cranes, pumps, and forklift trucks operated by the different terminals personnel within the port under the Valencia Port Authority domain.

Scope 3 is the one with more categories and subcategories. Additionally, it is the scope with less precision, as it is challenging to collect the data directly. In this case, the ship traffic category’s consumption and emissions were estimated using the direct traffic data and each type of ship’s consumption index based on technical sheets. The rest of the data was obtained from third-party companies’ different responses, although it was not possible to verify it directly.

However, as shown in Figure 2, it had a significant impact on the carbon footprint of the object of study, the Valencia Port, deserving a detailed analysis. Two categories stand out: ship traffic emissions and the electricity consumed by dealerships.

The carbon footprint associated with container ships’ traffic increased by 35.48% between 2008 and 2016, while the emissions associated with ferries’ traffic increased by 21.34%. The carbon footprint of the traffic of cruises and auxiliary tugs increased by 15.34% and 9.98%, respectively. However, the subcategory of “traffic of other ships” decreased by 15.86%. As this category represents the 20% in energy consumption of the ship traffic category, the result of the category implies an increase of only 20%. This value does not imply a significant growth as the activity rises 24.33% during the analysis period.

Regarding the electricity consumed by dealerships, the increase reached 29%. The subcategory of business-oriented activities represented 30.81%, and another rise of 18.76% responded to service-oriented activities. Only the subcategory of other activities decreased; in this case, 5.16%.

**Figure 7:**
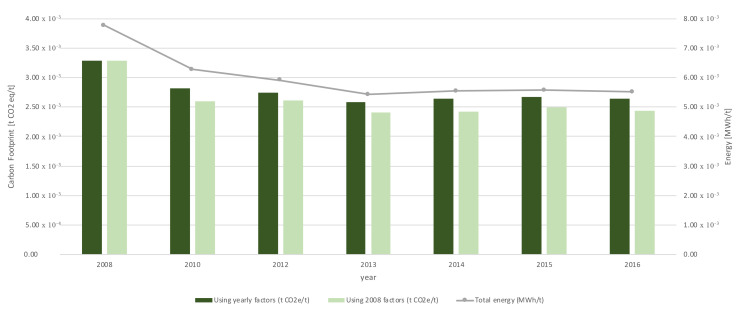
Results of complementary analysis: using constant factors (2008) and energy consumption.

A second assessment was made applying the emission factors of 2008 (first year of study) to all the period under analysis (Figure 7). The energy consumption [24] was also included in this assessment for comparative purposes.

The results of this second assessment are particularly interesting for the port authority and the port managers. Maintaining factors constant since the base year isolated the results of their initiatives and projects to improve the port’s environmental performance [24] from external factors that they cannot control as the region’s electrical mix or some political decisions regarding the consideration of categories. The influence of these external factors is evidenced in this figure.

The similarity between the evolution of energy consumption and the evolution of the carbon footprint is also interesting. Although the Port Authority of Valencia implemented several projects during the period assessed seeking environmental improvement, none of these projects included a change in the electricity source, a guarantee for a renewable energy source, or self-consumption projects [34]. This is why both variables were strictly linked, although, of course, they developed in different scales.

## 4. Conclusions

In recent years, the Port of Valencia has made a notable effort to reduce emissions generated by the port activity. Proof of this is that in the last six years, they managed to decrease their carbon footprint by 17% if emission factors in 2008 remained consistent during the assessment. The reduction was less than 1% if each year was assessed with their correspondent emissions factors. However, it was a good result considering that the port traffic had experienced a 24% growth during this period. Although significant improvements were made in the port, there was a wide margin for improvement in the activities’ sustainability.

Indirect emissions of machinery, vehicles, and ships, including electricity (scope 3), significantly impacted footprint results. Containers and cruise traffic was responsible for almost 40% of the CO_2_ eq emissions in this scope. The auxiliary engines of those ships that operate inside the port area had higher power than the rest of the equipment that influenced scope 3. The number of the ships was much greeter than the in-land machinery park and power of the port authority. This is why scope 3 was the main emitter.

The electrical machinery of terminals within the port was responsible for the greatest impact in scope 2 and lighting in buildings. As far as direct emissions in the infrastructures, machinery, and vehicles (scope 1) concern, diesel consumption overcome the impact of gasoline consumption. Gas had only a small effect on the last years.

Indirect emissions (scope 3) had the most significant impact, with 97% of the emissions, on average, during the period under analysis. This is why this scope should be the main focus of new policies seeking an improvement in the port authority’s environmental performance. Considering that the organization’s influence over the elements of scope 3 was not direct, implementing projects to improve minimization of the emissions of this scope was challenging.

Although the carbon footprint results obtained did not fit the methodology applied, where factors should be yearly updated, they are still beneficial for decision-making processes. Further studies should include the assessment of carbon footprint in different scenarios, for example, by replacing diesel with LNG.

## Figures and Tables

**Table 1:** Scopes and categories defined.

Scope	Category	Subcategory	Unit
1	Diesel consumption	-	l
	Gasoline consumption	-	l
	Gas consumption	-	kWh
2	Buildings	Lighting	kWh
		Air conditioning	kWh
	Stockyard and vial lighting	-	kWh
	Others	-	kWh
3	Diesel consumption	Business-oriented activities	l
		Service-oriented activities	l
		Other activities	l
	Gas consumption	Business-oriented activities	kWh
	Ship traffic	Container ships	Number of ships
		Cruise ships	Number of ships
		Ferris	Number of ships
		Other ships	Number of ships
		Auxiliary tugs	Number of tugs
	Transport within the freight terminal	-	km
	Dealership electricity	Business-oriented activities	kWh
		Service-oriented activities	kWh
		Other activities	kWh

## Appendix A

This appendix gathers the emissions factors applied for the study.

**Table A1:** Emission factors by categories for CO_2_ (kg/L). Main source [32].

Scope	Category and Subcategory	2008	2010	2012	2013	2014	2015	2016
1	Diesel	2.708	2.708	2.708	2.708	2.708	2.708	2.708
	Gasoline	2.295	2.295	2.201	2.205	2.205	2.205	2.205
	Gas	0	0	0	0	0	0.182	0.182
2	Building: Lighting	0.39	0.31	0.24	0.36	0.37	0.4	0.4
	Stockyard and vial lighting	0.39	0.31	0.24	0.36	0.37	0.4	0.4
	Building: Air-conditioning	0.39	0.31	0.24	0.36	0.37	0.4	0.4
	Others	0.39	0.31	0.24	0.36	0.37	0.4	0.4
3	Diesel: Business-oriented activities	2.708	2.708	2.708	2.708	2.708	2.708	2.708
	Diesel: Service-oriented activities	2.708	2.708	2.708	2.708	2.708	2.708	2.708
	Gas (1)	0.182	0.201799	0.201799	0.201799	0.201799	0.201799	0.201799
	Ship traffic: Containers (1)	Main engine: 0.6515 Auxiliary engine: 0.683						
	Ship traffic: Cruises (1)	Main engine: 0.6515 Auxiliary engine: 0.683						
	Ship traffic: Ferris (1)	Main engine: 0.6515 Auxiliary engine: 0.683						
	Ship traffic: Others (1)	Main engine: 0.6515 Auxiliary engine: 0.683						
	Ship traffic: Auxiliary tugs (1)	Main engine: 0.6515 Auxiliary engine: 0.683						
	Transport within the freight terminal	2.708	2.708	2.708	2.708	2.708	2.708	2.708
	Dealership electricity: Business-oriented activities	0.39	0.31	0.4	0.36	0.37	0.4	0.4
	Dealership electricity: Service-oriented activities	0.39	0.31	0.4	0.36	0.37	0.4	0.4
	Dealership electricity: Other activities	0.39	0.31	0.4	0.36	0.37	0.4	0.4

(1). Source [34].

**Table A2:** Emission factors by categories for CH4 (kg/kWh). Source [34].

Scope	Category and Subcategory	2008	2010	2012	2013	2014	2015	2016
1	Diesel	1.4 × 10^−5^	1.4 × 10^−5^	1.4 × 10^−5^	1.4 × 10^−5^	1.4 × 10^−5^	1.4 × 10^−5^	1.4 × 10^−5^
	Gasoline	1	1	1	1	1	1	1
	Gas	3.6 × 10^−6^	3.6 × 10^−6^	3.6 × 10^−6^	3.6 × 10^−6^	3.6 × 10^−6^	3.6 × 10^−6^	3.6 × 10^−6^
2	Building: Lighting	2.5 × 10^−6^	2.5 × 10^−6^	2.5 × 10^−6^	2.5 × 10^−6^	2.5 × 10^−6^	2.5 × 10^−6^	2.5 × 10^−6^
	Stockyard and vial lighting	2.5 × 10^−6^	2.5 × 10^−6^	2.5 × 10^−6^	2.5 × 10^−6^	2.5 × 10^−6^	2.5 × 10^−6^	2.5 × 10^−6^
	Building: Air-conditioning	2.5 × 10^−6^	2.5 × 10^−6^	2.5 × 10^−6^	2.5 × 10^−6^	2.5 × 10^−6^	2.5 × 10^−6^	2.5 × 10^−6^
	Others	2.5 × 10^−6^	2.5 × 10^−6^	2.5 × 10^−6^	2.5 × 10^−6^	2.5 × 10^−6^	2.5 × 10^−6^	2.5 × 10^−6^
3	Diesel: Business-oriented activities	1.4 × 10^−5^	1.4 × 10^−5^	1.4 × 10^−5^	1.4 × 10^−5^	1.4 × 10^−5^	1.4 × 10^−5^	1.4 × 10^−5^
	Diesel: Service-oriented activities	1.4 × 10^−5^	1.4 × 10^−5^	1.4 × 10^−5^	1.4 × 10^−5^	1.4 × 10^−5^	1.4 × 10^−5^	1.4 × 10^−5^
	Gas	3.6 × 10^−6^	3.6 × 10^−6^	3.6 × 10^−6^	3.6 × 10^−6^	3.6 × 10^−6^	3.6 × 10^−6^	3.6 × 10^−6^
	Ship traffic: Containers	Main engine: 0.000011 Auxiliary engine: 0.000008						
	Ship traffic: Cruises	Main engine: 0.000011 Auxiliary engine: 0.000008						
	Ship traffic: Ferris	Main engine: 0.000011 Auxiliary engine: 0.000008						
	Ship traffic: Others	Main engine: 0.000011 Auxiliary engine: 0.000008						
	Ship traffic: Auxiliary tugs	Main engine: 0.000011 Auxiliary engine: 0.000008						
	Transport within the freight terminal	1.4 × 10^−5^	1.4 × 10^−5^	1.4 × 10^−5^	1.4 × 10^−5^	1.4 × 10^−5^	1.4 × 10^−5^	1.4 × 10^−5^
	Dealership electricity: Business-oriented activities	2.5 × 10^−6^	2.5 × 10^−6^	2.5 × 10^−6^	2.5 × 10^−6^	2.5 × 10^−6^	2.5 × 10^−6^	2.5 × 10^−6^
	Dealership electricity: Service-oriented activities	2.5 × 10^−6^	2.5 × 10^−6^	2.5 × 10^−6^	2.5 × 10^−6^	2.5 × 10^−6^	2.5 × 10^−6^	2.5 × 10^−6^
	Dealership electricity: Other activities	2.5 × 10^−6^	2.5 × 10^−6^	2.5 × 10^−6^	2.5 × 10^−6^	2.5 × 10^−6^	2.5 × 10^−6^	2.5 × 10^−6^

**Table A3:** Emission factors by categories for N_2_O (kg/kWh). Source [34].

Scope	Category and Subcategory	2008	2010	2012	2013	2014	2015	2016
1	Diesel	1.4 × 10^−5^	1.4 × 10^−5^	1.4 × 10^−5^	1.4 × 10^−5^	1.4 × 10^−5^	1.4 × 10^−5^	1.4 × 10^−5^
	Gasoline	1	1	1	1	1	1	1
	Gas	3.6 × 10^−7^	3.6 × 10^−7^	3.6 × 10^−7^	3.6 × 10^−7^	3.6 × 10^−7^	3.6 × 10^−7^	3.6 × 10^−7^
2	Building: Lighting	5.07 × 10^−7^	5.07 × 10^−7^	5.07 × 10^−7^	5.07 × 10^−7^	5.07 × 10^−7^	5.07 × 10^−7^	5.07 × 10^−7^
	Stockyard and vial lighting	5.07 × 10^−7^	5.07 × 10^−7^	5.07 × 10^−7^	5.07 × 10^−7^	5.07 × 10^−7^	5.07 × 10^−7^	5.07 × 10^−7^
	Building: Air-conditioning	5.07 × 10^−7^	5.07 × 10^−7^	5.07 × 10^−7^	5.07 × 10^−7^	5.07 × 10^−7^	5.07 × 10^−7^	5.07 × 10^−7^
	Others	5.07 × 10^−7^	5.07 × 10^−7^	5.07 × 10^−7^	5.07 × 10^−7^	5.07 × 10^−7^	5.07 × 10^−7^	5.07 × 10^−7^
3	Diesel: Business-oriented activities	1.4 × 10^−5^	1.4 × 10^−5^	1.4 × 10^−5^	1.4 × 10^−5^	1.4 × 10^−5^	1.4 × 10^−5^	1.4 × 10^−5^
	Diesel: Service-oriented activities	1.4 × 10^−5^	1.4 × 10^−5^	1.4 × 10^−5^	1.4 × 10^−5^	1.4 × 10^−5^	1.4 × 10^−5^	1.4 × 10^−5^
	Gas	3.6 × 10^−7^	3.6 × 10^−7^	3.6 × 10^−7^	3.6 × 10^−7^	3.6 × 10^−7^	3.6 × 10^−7^	3.6 × 10^−7^
	Ship traffic: Containers	Main engine: 3.6 × 10^−5^ Auxiliary engine: 3.6 × 10^−5^						
	Ship traffic: Cruises	Main engine: 3.6 × 10^−5^ Auxiliary engine: 3.6 × 10^−5^						
	Ship traffic: Ferris	Main engine: 3.6 × 10^−5^ Auxiliary engine: 3.6 × 10^−5^						
	Ship traffic: Others	Main engine: 3.6 × 10^−5^ Auxiliary engine: 3.6 × 10^−5^						
	Ship traffic: Auxiliary tugs	Main engine: 3.6 × 10^−5^ Auxiliary engine: 3.6 × 10^−5^						
	Transport within the freight terminal	1.4 × 10^−5^	1.4 × 10^−5^	1.4 × 10^−5^	1.4 × 10^−5^	1.4 × 10^−5^	1.4 × 10^−5^	1.4 × 10^−5^
	Dealership electricity: Business-oriented activities	2.5 × 10^−6^	2.5 × 10^−6^	2.5 × 10^−6^	2.5 × 10^−6^	2.5 × 10^−6^	2.5 × 10^−6^	2.5 × 10^−6^
	Dealership electricity: Service-oriented activities	2.5 × 10^−6^	2.5 × 10^−6^	2.5 × 10^−6^	2.5 × 10^−6^	2.5 × 10^−6^	2.5 × 10^−6^	2.5 × 10^−6^
	Dealership electricity: Other activities	2.5 × 10^−6^	2.5 × 10^−6^	2.5 × 10^−6^	2.5 × 10^−6^	2.5 × 10^−6^	2.5 × 10^−6^	2.5 × 10^−6^

This appendix reports the input data used for the carbon footprint assessment by scope. The data source is the annual memories of the authority [31].

**Table A4:** Energy consumption for scope 1 in kWh. Source [31].

Category and Subcategory	2008	2010	2012	2013	2014	2015	2016
Diesel	491,754	477,180	497,802	421,342	408,149	367,920	336,702
Gasoline	391,200	391,776	313,274	311,186	226,628	241,176	239,986
Gas							74,925

**Table A5:** Energy consumption for scope 2 in kWh. Source [31].

Category and Subcategory	2008	2010	2012	2013	2014	2015	2016
Building: Lighting	2,475,441	2,533,209	2,470,830	1,947,924	2,291,604	2,688,823	3,309,970
Stockyard and vial lighting	815,135	931,076	1,361,472	1,691,063	1,726,301	2,025,532	2,493,452
Building: Air-conditioning	1,758,958	1,568,362	1,090,042	957,298	1,212,039	1,422,130	1,750,657
Others	14,348,356	5,947,697	4,233,146		3,775,225	2,047,206	1,320,876

**Table A6:** Energy consumption for scope 3 in kWh. Source [35].

Category and Subcategory	2008	2010	2012	2013	2014	2015	2016
Diesel: Business-oriented activities	156,645,968	127,942,305	132,210,151	112,941,714	113,283,157	122,465,919	121,392,432
Diesel: Service-oriented activities	4,512,170	4,429,032	4,628,324	4,197,734	4,210,431	5,173,962	5,523,957
Gas	42,678,242						
Ship traffic: Containers	65,179,562	80,481,812	79,428,691	73,822,114	79,232,468	80,692,970	88,305,890
Ship traffic: Cruises	2,667,136	3,001,279	3,620,179	3,641,971	3,218,228	2,953,239	3,077,725
Ship traffic: Ferris	5,578,318	3,992,083	4,213,405	5,063,270	4,539,293	6,415,669	6,769,348
Ship traffic: Others	25,041,144	22,588,369	22,224,802	20,217,055	32,416,833	24,758,886	21,071,067
Ship traffic: Auxiliary tugs	33,010,751	43,407,685	44,930,046	34,790,620	35,736,813	38,368,539	36,305,933
Transport within the freight terminal	73,131,420	66,084,288	70,722,884	72,621,853	69,415,324	74,279,372	76,978,166
Dealership electricity: Business-oriented activities	37,327,720	48,320,266	49,493,493	46,184,817	49,396,752	52,247,123	52,895,613
Dealership electricity: Service-oriented activities	1,104,442	1,419,682	1,553,712	1,585,647	1,586,003	1,482,932	1,420,833
Dealership electricity: Other activities	1,765,939	2,337,623	2,117,578	2,090,481	1,955,782	2,147,321	1,814,322
